# Acoustic Comparisons of Red Palm Weevil (*Rhynchophorus ferrugineus*) Mortality in Naturally Infested Date Palms after Injection with Entomopathogenic Fungi or Nematodes, Aluminum Phosphide Fumigation, or Insecticidal Spray Treatments

**DOI:** 10.3390/insects14040339

**Published:** 2023-03-30

**Authors:** Koko D. Sutanto, Mureed Husain, Khawaja G. Rasool, Richard W. Mankin, Abdalsalam O. Omer, Abdulrahman S. Aldawood

**Affiliations:** 1 Plant Protection Department, College of Food and Agriculture Sciences, King Saud University, P.O. Box 2460, Riyadh 11451, Saudi Arabia; 2 United States Department of Agriculture, Agricultural Research Service Center for Medical, Agricultural and Veterinary Entomology, P.O. Box 14565, Gainesville, FL 32607, USA

**Keywords:** date palm, insect acoustic activity, detection, entomopathogenic fungi and nematodes, integrated pest management

## Abstract

**Simple Summary:**

The efficacy of several treatments that could be used in integrated pest management (IPM) against infestations of red palm weevil (RPW) in date palm orchards was investigated. The effects of injections of entomopathogenic fungi and nematodes, aluminum phosphide, and emamectin, as well as fipronil sprays of trees in which naturally occurring RPW infestations had been identified were assessed using acoustic sensors to monitor mortality in naturally infested trees over a 6-month period after treatment. Reductions in insect activity levels in date palms monitored by acoustic sensors after treatments with entomopathogenic fungi and nematodes, emamectin, and aluminum phosphide indicated that each of these treatments caused significant RPW mortality.

**Abstract:**

Red palm weevil (RPW) management is important to the economic success of date palm agriculture. Monitoring with acoustic sensors was conducted in naturally infested trees in date palm orchards for six months after treatments with entomopathogenic fungi (*Beauveria bassiana* and *Metarhizium anisopliae*), entomopathogenic nematodes (*Steinernema carpocapsae*), aluminum phosphide, emamectin benzoate, or fipronil to evaluate their efficacy in an integrated pest management treatment vs. a distilled water injection. Reductions in the mean rates of RPW sound impulse bursts over time after treatment were used as indicators of RPW mortality. Entomopathogenic fungi and nematodes, aluminum phosphide, and emamectin benzoate were the most effective treatments, reducing RPW impulse burst rates within 2–3-months to levels indicating absence of infestation. However, when applied as a spray, fipronil had only a minor effect. The results indicate that treatments utilizing entomopathogenic fungi or nematodes can beneficially manage RPW in palm orchards and can help to limit treatments that may induce insecticide resistance or cause human and environmental harm. Furthermore, the use of an acoustic sensor can be beneficial in monitoring the activities of insect borers inside the tree trunk.

## 1. Introduction

The red palm weevil (RPW), *Rhynchophorus ferrugineus* (Olivier, 1790), is the most devastating palm tree pest globally [1]. It is difficult to detect in field environments because much of its lifecycle is concealed inside the host trunk [2]. After external signs of damage such as tunnels on the trunk, a fermented smell coming from the tunnels and frass at the tunnel openings [3] begin to appear, at which point many trees already have suffered irreparable harm. In Saudi Arabia, strategies to detect RPW in palm orchards have included inspections of palm trunks and pheromone trapping [4,5]. Strategies to manage RPW have included insecticides [6,7,8,9], and treatments with entomopathogenic organisms such as nematodes [10,11], bacteria [12,13], viruses [14,15], and fungi [16,17,18,19,20]. Deep-learning techniques have been developed to distinguish RPW images from those of other insects found inside palm trees [21]. In addition, an optical sensor was used in a recent study to detect RPW presence inside a date palm tree [22]. However, such detection and management strategies have been only partially successful in restricting RPW from spreading to areas with healthy palm trees [21].

Acoustic sensors can detect the presence of RPW activities inside the trunk before infestations are discovered by trunk inspections or pheromone traps [23,24,25]. Acoustic sensors enable in situ monitoring of RPW larval activities and development through different instars [20] and their use is less damaging to palm trees than many other RPW detection methods. Opportunities to apply acoustic sensors to monitor or enhance the effectiveness of treatments against RPW are only in the beginning stages of investigation.

In recent years, acoustic sensors have been used successfully in numerous studies to detect and monitor the presence of RPW inside palm trees [20,23,24,25,26]. A cloud-integrated, micro-computer-based bioacoustics monitoring system was developed for IPM in agriculture [25]. Methods were established to identify features of RPW feeding and movement sounds in palm trunks that distinguish them from background sounds [23,24,26,27,28]. The signal processing system DAVIS [25] first calculates individual spectra of each sound impulse in a recording, compares each spectrum with spectral profiles (means) of sound impulses known to be produced by RPW, and then examines the temporal pattern in trains (bursts) of sound impulses that match the RPW profiles to estimate the likelihood that sounds detected from a palm tree indicate the presence of an RPW infestation [29].

The primary objective of this study was to evaluate the efficacy of different treatments that could be used in an IPM program against RPW. To evaluate the efficacy of the treatments, unmanaged, naturally occurring infested date palm orchards were selected and an acoustic sensor was used to monitor the RPW mortality associated with various treatments.

## 2. Materials and Methods

### 2.1. Study Site and Tree Selection

The field study was conducted in Huraymila (25°07.284″ N, 46°07.021″ E) Riyadh, Saudi Arabia. The selection of date palm trees was based on size, between 1–5 m height; on visual symptoms, including tunnels on the trunk and at the base of the date palm fronds that contained chewed plant tissue with a fermentation odor and seeped a thick brown liquid substance; and on confirmation of RPW sound production with the use of an acoustic sensor, TreeVibes (Insectronics Insect Surveillance Technology, Chania, GR–73100, Crete, Greece). Each treatment or inert, distilled water injection was assessed separately on 6–10 trees with the mean of all monthly measurements on one tree serving as one replicate.

### 2.2. Treatments and Inert Control Injections

The tested treatments were: (1) injections of entomopathogenic fungi (*Beauveria bassiana* and *Metarhizium anisopliae*) that were previously screened in the lab and isolated from red palm weevil/Coleoptera from Saudi Arabia [16], (2) a mix of both fungi, (3) an entomopathogenic nematode, *Steinernema carpocapsae* (Palmanem, Koppert Biological System, Berkel en Rodenrijs, The Netherlands), (4) sprays of the chemical insecticide fipronil (Fiprol, Asian Crop Care, Vijayawada, Andhra Pradesh, India), (5) fumigation with phosphine (aluminum phosphide), and (6) micro-injection of TreeCare emamectin benzoate (Revive/Aretor 4EC, Syngenta, Basel, Switzerland). Distilled water was used as an inert control injection. The entomopathogenic fungi, nematodes, and the inert control were injected using balloons, with one balloon per meter of tree height. Fipronil was sprayed as in [30,31]. Aluminum phosphide was applied as tablets after drilling the trunk, and then the trunk was wrapped with plastic sheets [32,33,34]. A micro-injector was used for the TreeCare emamectin benzoate treatments.

### 2.3. Fungal Isolate Mass Production

Fungal isolates that grew successfully on PDA medium were selected for mass production on wheat medium [35,36]. First, wheat was washed in regular water then rinsed with distilled water. Second, before being autoclaved, 1 kg of wheat was kept overnight in a plastic container. A small piece of each fungal isolate (1 cm) was inoculated on autoclaved wheat medium. For two to three weeks, inoculated wheat was kept in the dark at temperatures of 28 ± 1 °C and relative humidity (RH) of 80 ± 5%. After germination, the wheat medium was moved to a sterile plastic box and dried in an incubator. Fungi were dried out and powdered.

The fungi were mass-produced to create fungal solutions for injection into date palm trunks. The concentration of fungi was determined using a hemocytometer and a microscope. The concentration in this experiment, 1 × 10^9^ conidia/mL, was the same as in a previous study conducted under laboratory conditions [16]. Fungi were held in 5-l buckets.

### 2.4. Application of Entomopathogenic Fungi and Inert Control

A balloon injector (Ynject, Cordoba, Spain) was filled with 100 mL of laboratory-screened fungal solutions by applying five consecutive injections from a 20-mL syringe (Henke–Sass Wolf GmBH, Tuttlingen, Germany) to deliver the fungal isolates into selected date palm trees. The two fungal isolates (*B. bassiana* and *M. anisopliae*) and their 50:50 mix were applied at doses up to 400 mL/tree, depending on the tree height.

Injection points were marked around each date palm trunk to drill holes at a 30-degree angle using an 8 mm diameter brad-point drill bit. Four spiral holes were drilled around the trunk at 25, 50, 75, and 100 cm above ground. The trunk received fungal solution immediately after drilling. The dosage was proportional to tree height: a 4–5 m tree received 4 balloons (400 mL/tree), and a 1 m tree received 1 balloon (100 mL/tree), etc. (Table 1). Each hole received 100 mL fungal solution from a balloon. For inert control tests, date palm trees were injected with distilled water. The time required for the injection process of each balloon was 2–10 min. All infested date palm trees were injected on the same day.

Each treatment was tested separately on 6–10 trees, with one tree serving as one replicate. Monthly observations and acoustic measurements were conducted after treatment.

### 2.5. Application of Entomopathogenic Nematodes

Nematodes were injected into the date palm at the recommended dose. The Koppert package (250 million nematodes/70 gm) was used to make the nematode suspension, and 3 g of nematodes were diluted in 400 mL of water to obtain 10 million nematodes. In 100 mL of water, each balloon contained 2.5 million nematodes. The dosage was determined based on tree height: a 4–5 m tree received 4 balloons (full dose), and a 1 m tree received 1 balloon. Each balloon injection process took 2–10 min (depending on the size of the tree).

The nematode solution was delivered into the trunk via balloon injector probe after drilling 8 mm diam. holes at a 45-degree angle. During injection, the probe was fully inserted and then slightly moved outward to allow space and air for nematodes to be delivered smoothly into the date palm trunk. This treatment was replicated ten times (one tree was representative of one replicate).

### 2.6. Application of Chemical Insecticides

#### 2.6.1. Fipronil (Fiprol)

Fipronil (Delta, Saudi Arabia) was applied with a sprayer (Albaitarie, Huraymila, Saudi Arabia). The fipronil solution was prepared by diluting 250 mL of fipronil into 250 L of water and mixed well in the spray tank. A 50-l insecticide solution was applied to one tree. The spraying process took approximately 3–4 min.

#### 2.6.2. Phosphine (Aluminum Phosphide)

Phosphine treatment was applied using a plastic sheet wrapper (fumigation) provided by the Ministry of Environment, Water and Agriculture (Huraymila, Saudi Arabia). The date palm fronds were removed before applying the phosphine tablets. Four holes were drilled in a spiral manner with a drill machine. A single aluminum phosphide tablet (56% Celphos; Lahore, Pakistan) was inserted into each hole and the holes were closed with mud. Two sponges were placed on each tree, one on the bottom and one on the top, and then the tree was covered with a plastic sheet and tightly wrapped. Each insecticide tablet has a half-life of 2.5 h, so the tree must be wrapped on all sides. The wrapping process took between 25 and 30 min (depending on the size of the tree). After one week, the plastic wrapping was removed from the trees.

#### 2.6.3. TreeCare (Emamectin Benzoate)

TreeCare (Syngenta, Greensboro, NC, USA) treatment was applied using a 10.5 mL micro-injector [7]. The date palm fronds were cleaned before applying the insecticide to ensure proper injection. Four holes (one in each direction) were drilled at the base of the tree. In total, 42 mL of insecticide was injected into each tree. The injection process took 1–1.5 min, and each hole was then sealed with a green plug.

### 2.7. Field Evaluation Using Acoustic Sensor: TreeVibes

A TreeVibes device (Insectronics, Greece) was used to determine the effectiveness of various treatments against RPW infestation by determining the decrease in RPW activity inside the date palm trunk over time after treatment. The device outputs data to a server/cloud system so that feature identification and another signal processing can be performed. To help assess the efficacy of all treatments in comparison to natural infestation, acoustic recordings also were obtained monthly from 6 date palms that were determined to have been naturally infested in the field.

#### Acoustic Recordings

Date palms selected for recordings were marked once on each trunk, 1 m above ground level. Holes (30-degree angle) were inserted to a 35 cm depth at each mark with a drill machine equipped with a brad-point drill bit (diameter, 8 mm). It was previously confirmed that RPW signals can be detected over 1–4 m distances using such methods [29]. The TreeVibes recording device includes a 35 cm probe (waveguide) that is inserted into the tree hole. It is equipped with headphones that enable the user to assess the sound signal produced by RPW activities as the recordings are collected. The sound signals were transmitted to the server cloud and subsequently were analyzed using the DAVIS program [24], which identified and counted acoustic features such as the rate of production of insect sound impulse bursts, *r_s_*, i.e., the number of bursts produced per second, that can be used as indicators of the likelihood of infestation and, when monitored over time, can be used to estimate reductions in infestation likelihood [27,37]. The late instars of RPW larvae also produce distinctive squeals [38]. that were detected and analyzed as individual RPW sound bursts. Previous studies determined that trees in which values of *r_s_* < 0.02 were detected had a low likelihood of infestation. Higher rates of bursts, 0.02 ≥ *r_s_* < 0.06, indicated greater (medium) likelihood of infestation, and recordings at rates of *r_s_* ≥ 0.06 almost always indicated the presence of RPW infestation [38]. Consequently, if the burst rate measured in a date palm indicates high likelihood of RPW infestation at the beginning of treatment but later decreases to low likelihood of infestation after further assessments, the treatment of that tree can be estimated to have eliminated its infestation [38].

For this experiment, recording parameters for each tree were set to include separate files of 20 s duration collected over a 5 min period, i.e., a total of 100 s of signal were collected from each tree, each month. Time stamps of each recording were provided as the recorded data were sent to the server cloud. Subsequently, the sound data were downloaded manually and stored on a PC computer (Dell, OPTIPLEX 7010) in the laboratory. In the initial stage, Raven Pro 1.5 [39] was used to screen the signals in subsequent analyses of RPW burst rates by DAVIS. Monthly comparisons were made of the differences in burst rates between treated and untreated date palm trees. During the recording process, precautions were taken to minimize the background noise, such as avoiding any contact between the sensor and date palm leaves or other surfaces [26].

### 2.8. Statistical Analysis

The experiments were carried out in a completely randomized block design. For statistical comparisons, the rates of impulse bursts, *r_s_*, were compared with analysis of variance using SAS and the means were separated using Tukey’s test at *p* < 0.05 [40]. Although the experimental palms were known to be infested due to the presence of visual symptoms, the initial numbers and ages of RPW adults and larvae in each palm were unknown, and previous studies have indicated these strongly affect the mean impulse burst rate [20,26,29]. Consequently, the mean magnitude of *r_s_* in a particular treatment serves primarily as an indicator of the likelihood of continued infestation after treatment. Magnitudes of *r_s_* > 0.02/s indicate a medium or high likelihood of the presence of infestation [38]. Each tree would be considered to have an initially unknown level of infestation and might be infested by additional RPW after treatment. Successful treatment would require that the treatment provide residual control over the 6-month period of monitoring so that the measured rates of RPW impulse bursts would decrease to and remain at low likelihood of infestation.

## 3. Results

### 3.1. Monthly Impulse Burst Rates (r_s_) Monitored from Infested Date Palm Trees after Treatments

The mean monthly impulse burst rates of RPW sound activities within the infested tree are listed for all treatments over a 6-month period (Table 2). Means in each treatment were significantly different from the injection-control mean after the second month of observation except at the sixth month when four of six injection-control trees died. Mortality occurred only in the trees injected with inert ingredients. As noted in [38], the occurrence of impulse burst rates > 0.02/s indicates medium or high likelihood that a RPW infestation is present, which suggests that all the treatments with active ingredients had successfully eliminated RPW infestations within five months.

### 3.2. Evaluation of the Rates of Impulse Bursts (r_s_) Generated from the Infested Date Palm Trees after Treatments

**Figure 1:**
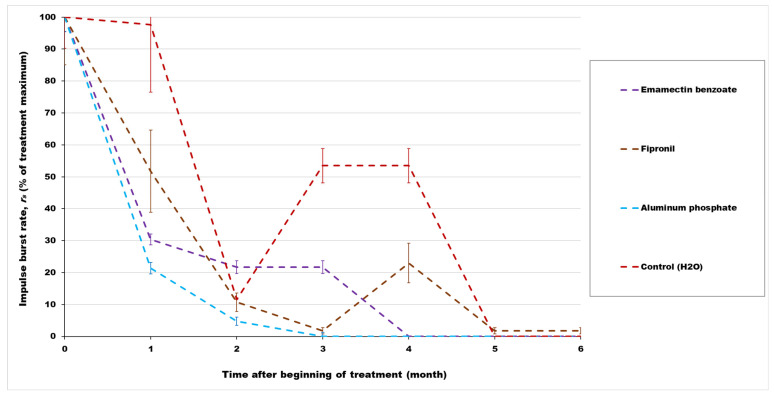
Mean impulse burst rates within RPW-infested date palm trees as a percentage of the maximum mean obtained from a given chemical treatment on a time scale beginning at the time of treatment (0) and then at specified months thereafter. Bars indicate the standard error of the mean percentage in each chemical treatment or distilled water control during the specified month.

**Figure 2:**
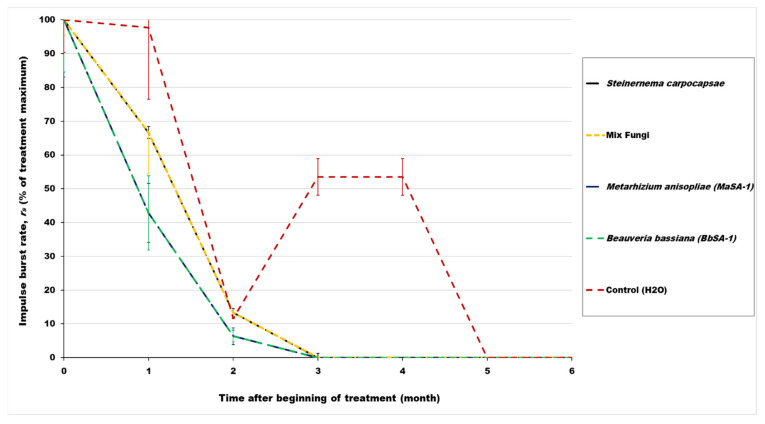
Mean impulse burst rates within RPW-infested date palm trees in each biological control treatment as a percentage of the maximum of all means for that treatment on a time scale beginning at the time of treatment (0) and then at specified months thereafter. Bars indicate the standard error of the mean percentage in each biological control treatment or distilled water control during the specified month.

To compare effects of the different treatments on RPW activity over time on the same scale, the scale for all treatments was set to range from 0 to 100% of each treatment maximum in Figure 1 and Figure 2. Activity in the aluminum phosphide, *B. bassiana*, and fungal treatments was variable due to the unknown initial magnitude of RPW infestation and the natural variability of insect behavior rhythms, but the mean impulse burst rates decreased to zero within three months and remained negligible thereafter. Similarly, activity in the emamectin benzoate treatment was initially variable, but declined to zero within four months.

Mean RPW impulse burst rates in the fipronil treatments and water controls remained above a previously identified 0.02 impulse bursts/s threshold for medium likelihood of RPW infestation [38] through the fourth month after treatment (Table 2). However, because four of six control trees unexpectedly suffered mortality before the end of testing, the mean impulse burst rates in the controls, as well as in the entomopathogenic fungal or nematode injection, phosphine fumigation, and insecticidal spray treatments, decreased to levels indicating low likelihood of infestation within six months after the beginning of the experiment. After the fourth month, the mean RPW impulse burst rate in the fipronil treatment also decreased to levels indicating low likelihood of infestation.

## 4. Discussion

The treated date palm trees, as well as those in the distilled water control, produced varying rates of RPW sound impulse bursts that were summarized in monthly averages. As expected, the mean RPW impulse burst rates of date palms receiving only the distilled water injections were significantly higher than in treated trees by months 3–4, leading to collapse and death of control trees after five months. Activity in the aluminum phosphide, emamectin benzoate, *B. bassiana*, and fungal treatments decreased rapidly to zero within three months and remained negligible thereafter. After fipronil treatment, the rates of RPW impulse bursts declined more slowly than in the other treatments with active ingredients above, but ultimately reduced to zero after five months.

The results for aluminum phosphide fumigation was supported by other studies showing that ten tablets of aluminum phosphide inserted in the trunk of a date palm killed all stages of the RPW, with 23–43 individuals per tree present when the trunk was dissected [32]. It is possible to fumigate RPW-infested date palm trees with aluminum phosphide, and the authors recommend it as a very effective treatment against RPW [41]. However, the sustained use of aluminum phosphide can increase RPW resistance to phosphine by a factor of 79 [9].

Injections of emamectin benzoate into the date palm trunk significantly reduced RPW impulse burst rates to zero after four months. This finding was supported by other research, which found that injecting emamectin benzoate killed more than 90% of RPW individuals [42]. In another study, emamectin benzoate injected into date palm trees artificially infested with RPW remained effective for 150 days [43].

This study is supportive of other studies that evaluated the effectiveness of microorganisms [44] and fungal treatments against RPW [26,45]. RPW can be managed by microorganisms that cause infection in insects [46]. In another study, RPW movement and feeding activities in date palm trees produced significantly lower impulse burst rates [20] after treatment with *B. bassiana*. It is possible that infected RPW larvae within date palm trees become weaker, lose their appetite, and move more slowly [44,47]. It should be noted that spores of fungal isolates with weak germination, low pathogenicity, and low water solubility are not recommended for use against the RPW [2]. Likewise, after the application of entomopathogenic nematode injections into date palm trees, impulse burst rates were significantly reduced and declined to zero after four months. Other studies have obtained similar results [11,48].

The date palms used in this study were naturally infested. Therefore, the numbers and ages of the RPW larvae and adults living within the palm trunks could not be determined; nor was it known whether multiple generations of adults and larvae were present, which may have contributed to the mortality of the control trees that died during this study. Our findings were consistent with other reports that it may not be possible to determine the age of RPW larvae using acoustic devices due to the large variation of sound rates produced by different behaviors, and the strong effects of the distance between the larva and the acoustic detector on the detectability of acoustic signals [20]. However, due to the large sizes of the RPW larvae and adults, the sound signals they produce within date palm trees can be detected reliably enough with acoustic devices to confirm the presence or absence of RPW infestation with sufficient precision for many purposes [26,28,29,38,49,50]. Similarly, acoustic sensors detected RPW larval activities in infested coconut palm trees at frequencies of 500–1000 Hz, but no sound signal was detected from uninfested trees [51]. RPW-infested trees exhibited a distinct spectral profile because different insect borers have distinctive movement and feeding activity patterns that can be differentiated relatively easily from the temporal patterns of background noise impulses [52].

In this study, a goal was to explore the use of acoustic devices to determine the efficacy of different treatments against RPW under field conditions because palm dissection is a very laborious, time-consuming, expensive, and destructive method for assessing treatment effectiveness in the field [20]. Uses of acoustic sensors to evaluate the efficacy of fungal treatments against RPW have also been reported from Malaysia [20,26]. Acoustic sensors can detect insect borers such as RPW inside trees of other palm species: date palm [26,50], canary palm [49,50,52,53], coconut palm [28,51], and oil palm [28]. There is opportunity to evaluate the efficacy of treatments against RPW and other borers in these palm species as well. It should be noted also that similar studies have been performed to monitor the decline of stored product insect activity and increase in mortality after grain was placed in hermetic storage bags, e.g., [54]. One difference in the signal analyses of the stored product insect study is that the activity patterns were different, which required that different spectral profiles be used for the operation of the DAVIS signal analysis program.

## 5. Conclusions

The treatments described herein can manage RPW populations in treated palm trees and reduce its further spread in areas currently free of infestation for at least a five-month period. Fumigation and injections of several treatments used in the present study, including aluminum phosphide, emamectin benzoate (TreeCare), *Beauveria bassiana*, *Metarhizium anisopliae*, and *Steinernema carpocapsae* were found to be very promising against RPW. These findings unequivocally demonstrate that acoustic sensors can be exploited to monitor RPW infestation for timely management of this ruinous pest of date palm trees worldwide.

## Figures and Tables

**Table 1:** Fungal injection procedures.

Fungi	Tree Group	Tree Height (m)	Balloon/Tree	Total Volume, Conidia/mL/Balloon
*Beauveria bassiana*/ *Metarhizium anisopliae*/ Mixed fungi	1	<1	1	100 mL, 1 × 10^9^ conidia/mL
	2	1–1.9	2	
	3	2–2.9	3	
	4	3–3.9	4	
	5	4–5	4	

Note: the injections were spaced spirally in 1 m increments around the tree, beginning 25 cm above the ground.

**Table 2:** Impulse burst rate means of red palm weevil sound activities within infested trees for all treatments during a six-month period.

Treatment	Mean monthly Impulse Burst Rates (*r_s_*) of Red Palm Weevil (RPW) Sound Activities within Initially Infested Palm Trees							Statistical Analysis F_df1,df2_; *p*
	0	1	2	3	4	5	6	
Control (water)	0.43 ± 0.09 aAB	0.42 ± 0.21 abA	0.05 ± 0.01 abA	0.23 ± 0.05 abA	0.23 ± 0.05 abA	0 ± 0 bA	0 ± 0 bA	F_6,35_ = 2.99; <0.02
Fipronil (Fiprol)	0.56 ± 0.14 aA	0.29 ± 0.12 abAB	0.06 ± 0.02 bA	0.01 ± 0.01 bB	0.13 ± 0.06 bB	0.01 ± 0.01 bB	0.01 ± 0.01 bB	F_6,35_ = 6.77; <0.001
Fungus (*B. bassiana*)	0.52 ± 0.15 aAB	0.22 ± 0.11 abAB	0.03 ± 0.01 bA	0 ± 0 bB	0 ± 0 bC	0 ± 0 bA	0 ± 0 bA	F_6,42_ = 7.66; <0.001
Fungus (*M. anisopliae*)	0.55 ± 0.16 aA	0.11 ± 0.08 bB	0.03 ± 0.02 bA	0 ± 0 bB	0 ± 0 bC	0 ± 0 bA	0 ± 0 bA	F_6,42_ = 7.85; <0.001
Mixed fungal isolates	0.28 ± 0.04 aAB	0.18 ± 0.12 abAB	0.03 ± 0.01 bcA	0 ± 0 bB	0 ± 0 bC	0 ± 0 bA	0 ± 0 bA	F_6,56_ = 5.23; <0.001
TreeCare (emamectin benzoate)	0.23 ± 0.04 aB	0.07 ± 0.01 bB	0.05 ± 0.02 bA	0.05 ± 0.02 bB	0 ± 0 bC	0 ± 0 bA	0 ± 0 bA	F_6,70_ = 14.61; <0.001
Aluminum phosphide	0.35 ± 0.09 aAB	0.07 ± 0.04 bB	0.01 ± 0.01 bA	0 ± 0 bB	0 ± 0 bC	0 ± 0 bA	0 ± 0 bA	F_6,42_ = 10.29; <0.001
Nematode (*S. carpocapsae*)	0.24 ± 0.04 aAB	0.11 ± 0.01 bB	0.03 ± 0.01 cA	0.03 ± 0.01 cB	0 ± 0 cC	0 ± 0 cA	0 ± 0 cA	F_6,70_ = 21.51; <0.001
Statistical analysis (F_7,56_; *p*)	F = 2.19; 0.052	F = 1.51; 0.1878	F = 0.54; 0.7982	F = 14.07; <0.0001	F = 14.70; <0.0001	F = 27.35; <0.0001	F = 1.56; 0.1700	

Means that are followed by the same small letter in the same row or by the same capital letter in the same column are not significantly different at *p* < 0.05.

## Data Availability

All relevant data are given in the paper.
